# Pharmacodynamic Modeling of Cell Cycle Effects for Gemcitabine and Trabectedin Combinations in Pancreatic Cancer Cells

**DOI:** 10.3389/fphar.2016.00421

**Published:** 2016-11-15

**Authors:** Xin Miao, Gilbert Koch, Sihem Ait-Oudhia, Robert M. Straubinger, William J. Jusko

**Affiliations:** ^1^Department of Pharmaceutical Sciences, University at Buffalo, State University of New YorkBuffalo, NY, USA; ^2^Pediatric Pharmacology and Pharmacometrics, University of Basel, Children's HospitalBasel, Switzerland; ^3^Department of Pharmaceutics, Center for Pharmacometrics and Systems Pharmacology (Orlando), College of Pharmacy, University of FloridaOrlando, FL, USA

**Keywords:** cell cycle, pancreatic cancer, drug combination, pharmacodynamic models, gemcitabine, trabectedin

## Abstract

Combinations of gemcitabine and trabectedin exert modest synergistic cytotoxic effects on two pancreatic cancer cell lines. Here, systems pharmacodynamic (PD) models that integrate cellular response data and extend a prototype model framework were developed to characterize dynamic changes in cell cycle phases of cancer cell subpopulations in response to gemcitabine and trabectedin as single agents and in combination. Extensive experimental data were obtained for two pancreatic cancer cell lines (MiaPaCa-2 and BxPC-3), including cell proliferation rates over 0–120 h of drug exposure, and the fraction of cells in different cell cycle phases or apoptosis. Cell cycle analysis demonstrated that gemcitabine induced cell cycle arrest in *S* phase, and trabectedin induced transient cell cycle arrest in *S* phase that progressed to *G*_2_/*M* phase. Over time, cells in the control group accumulated in *G*_0_/*G*_1_ phase. Systems cell cycle models were developed based on observed mechanisms and were used to characterize both cell proliferation and cell numbers in the *sub G*_1_, *G*_0_/*G*_1_, *S*, and *G*_2_/*M* phases in the control and drug-treated groups. The proposed mathematical models captured well both single and joint effects of gemcitabine and trabectedin. Interaction parameters were applied to quantify unexplainable drug-drug interaction effects on cell cycle arrest in *S* phase and in inducing apoptosis. The developed models were able to identify and quantify the different underlying interactions between gemcitabine and trabectedin, and captured well our large datasets in the dimensions of time, drug concentrations, and cellular subpopulations.

## Introduction

Pancreatic cancer is a highly aggressive malignancy and shows resistance to almost all existing treatments (Oberstein and Olive, 2013). Gemcitabine (GEMZAR, Eli Lilly, Indianapolis, IN), a standard therapy for the treatment of advanced pancreatic cancer, can disrupt DNA replication and activate the *S* phase checkpoint (Yip-Schneider et al., 2001; Morgan et al., 2005; Robinson et al., 2006) However, the benefits of gemcitabine monotherapy are limited, and combinations of other agents with gemcitabine may improve survival of pancreatic cancer patients. Trabectedin (YONDELIS®, Et-743; Johnson and Johnson Pharmaceutical Research and Development, Raritan, NJ, USA; PharmaMar S.A.U., Madrid, Spain) is a promising anticancer agent that has demonstrated clinical activity in many drug-resistant cancer cell lines, and has been approved by the US Food and Drug Administration for advanced soft tissue sarcoma. It has three tetrahydroisoquinoline rings. The A and B subunits bind covalently to the DNA minor groove and bend DNA toward the major groove, and the C ring protrudes to interact with adjacent macromolecules such as transcription factors (D'Incalci and Galmarini, 2010). Trabectedin was found previously to cause cell cycle arrest at *S* and *G*_2_/*M* phases in many human tumor cell lines (Gajate et al., 2002; Simoens et al., 2003). Because of its unique mechanisms of action (D'Incalci and Galmarini, 2010), trabectedin has been reported to exert anti-tumor activities in many malignancies, including soft-tissue sarcomas, ovarian carcinomas, and breast cancer (D'Incalci et al., 2002; D'Incalci and Zambelli, 2015).

Our previous report provided indications from the literature that gemcitabine and trabectedin have mechanisms that may interrelate to produce synergism in their chemotherapeutic effects and we demonstrated that the combination of gemcitabine and trabectedin exerts synergistic cytotoxic effects on pancreatic cancer cells (Miao et al., 2016a). Here we have extended the work, assessing cell cycle subpopulations in two pancreatic cancer cell lines to examine drug interactions, because asynchronous cancer cell cultures are composed of different subpopulations, and each may have different sensitivities to drugs. Previously we also developed a pharmacodynamic (PD) model that was able to characterize simultaneously 32 sets of data for single-agent and combined drug effects on pancreatic cancer cell lines (Miao et al., 2016a). Here we have expanded the model to integrate additional data regarding the temporal changes of cell numbers in *sub G*_1_, *G*_0_/*G*_1_, *S*, and *G*_2_/*M* phases, so as to determine how each subpopulation contributes to the observed effects of the drugs, as single agents or combined.

Cell cycle models have been developed previously to characterize cell cycle arrest and induction of apoptosis for drugs such as gemcitabine (Jusko, 1973; Hamed et al., 2013; Zhu et al., 2015). In this study, we extended a cell cycle model (Hamed et al., 2013) to integrate components of our previous model (Miao et al., 2016a) in order to characterize cell cycle effects of drug combinations. We measured cell proliferation as temporal changes in total cell numbers, as well as the fraction of cells in each phase of the cell cycle, and used the absolute cell number in each cell cycle phase as a PD endpoint for model fitting and qualification. The cell cycle models feature the dimensions of time, drug concentration, and drug effects on cell subpopulations. The application of mathematical modeling of cell subpopulation responses to combination therapy, and gaining an understanding of drug effects upon the transition rates between cell cycle phases, provides a greater insight into the molecular mechanisms underlying the synergistic effects of gemcitabine and trabectedin.

## Materials and methods

### Reagents

Gemcitabine hydrochloride was purchased from Eli Lilly (Indianapolis, IN), dissolved in sterile double-distilled water, and stored at −20°C at a stock concentration of 50 mM. Trabectedin, obtained as a gift from PharmaMar (Madrid, Spain), was prepared as a 1 mM stock solution in dimethylsulfoxide (DMSO) and stored at −20°C.

### Cell culture

The pancreatic cancer cell lines MiaPaCa-2 and BxPC-3 were purchased from American Type Culture Collection (ATCC). MiaPaCa-2 cells were grown in DMEM (Cellgro, Manassa, VA) supplemented with 10% (v/v) fetal bovine serum (Cellgro). BxPC-3 cells were cultured in RPMI (Cellgro), 10% (v/v) fetal bovine serum and 1% (v/v) sodium pyruvate (Gibco, Grand Island, NY). Cells were maintained in 5% CO_2_ at 37°C with 95% humidity and grown as monolayers in T75 tissue culture flasks (BD Biosciences, Bedford, MA).

### Cell proliferation assay

To enable exponential cell growth for the duration of the experiment, 1.5 − 2 × 10^6^ cells in 5 mL fresh medium were seeded in 6-well plates and allowed to adhere overnight. Cells then were treated in triplicate with 4 different concentrations of gemcitabine and trabectedin, alone or combined, for 6 different time intervals of up to 120 h (Table 1). Equivalent volumes of water or DMSO were added as vehicle controls for the two drugs. At the appropriate times, triplicate wells of cells were washed twice with Dulbecco's phosphate buffered saline (Gibco) and suspended by incubating in 500 mL 1 × Trypsin EDTA (Cellgro) for 5 min. Cells were counted using a Coulter Counter model Z2 (Beckman Coulter, Hialeah, FL), and analyzed by flow cytometry.

**Table 1:** **Concentrations of gemcitabine (G) and trabectedin (T) tested as single agents and in combination in cell culture studies**.

**Cell line**	**Drug**	**Concentration (nM)**
MiaPaCa-2	Gemcitabine (G)	0, 15, 23, 45
	Trabectedin (T)	0, 0.8, 1, 1.5
	Combinations	15G+0.8T, 23G+0.8T, 23G+1T, 45G+1T
BxPC-3	Gemcitabine (G)	0, 11, 17, 34
	Trabectedin (T)	0, 0.5, 0.7, 1.1
	Combinations	11G+0.5T, 17G+0.5T, 17G+0.7T, 34G+1.1T

### Cell cycle assay

Aliquots of the cell suspensions were fixed in 70% cold ethanol (Decan Laboratories, King of Prussia, PA) and stored at −20°C until flow cytometry analysis, which was performed within 2 weeks. In brief, the ethanol was removed and cells were washed in cold Stain Buffer (BD Pharmingen, San Diego, CA) and treated for 30 min at room temperature in the dark with propidium iodide (PI) containing RNase (BD Pharmingen). Based upon analysis of the DNA content of each cell, histograms of cell distribution in the different cell cycle phases were obtained using a FACSort flow cytometer (Becton Dickinson, San Jose, CA). The CellQuest, WinList, and ModFit LT 4.0 software (Verity Software, Topsham, ME) were used for analysis of cell cycle distribution and determination of the fraction of cells in the *G*_0_/*G*_1_, *S*, and *G*_2_/*M* phases. Apoptosis was measured by quantifying the *sub G*_1_ peak. Measurement accuracy was evaluated from the coefficient of variation (CV%). Each sample was assayed in triplicate. The number of cells in each cell cycle phase was obtained by multiplying the total cell numbers of each sample by the fraction of cells in each phase.

### Mathematical model

**Figure 1:**
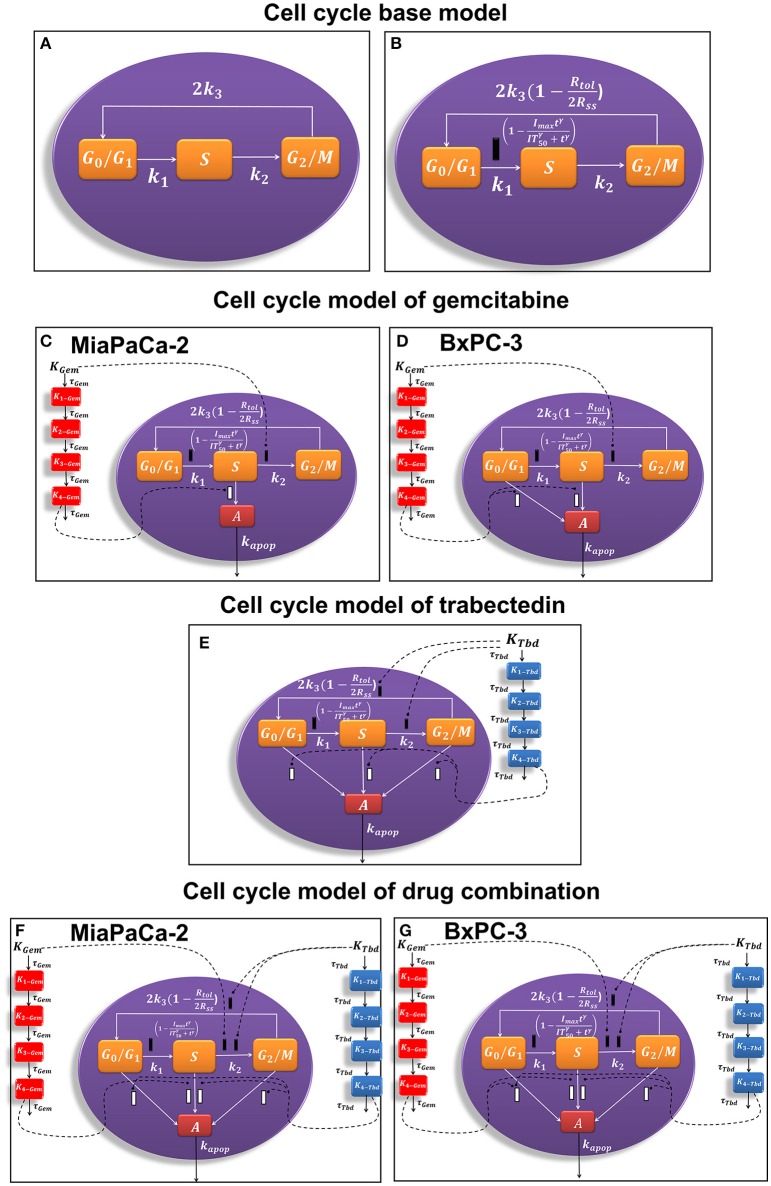
**Schematic of the pharmacodynamic models for single and combination drug effects on the cell cycle**. **(A)** Cell cycle base model. **(B)** Alternative cell cycle base model. **(C)** Cell cycle model of gemcitabine for MiaPaCa-2 cells. **(D)** Cell cycle model of gemcitabine for BxPC-3 cells. **(E)** Cell cycle model of trabectedin for MiaPaCa-2 and BxPC-3 cells. **(F)** Cell cycle model of drug combination for MiaPaCa-2 cells. **(G)** Cell cycle model of drug combination for BxPC-3 cells.

The models were premised on known cell cycle processes with components informed by observations of perturbations caused by the drugs. Mathematical models (Figure 1) were developed based on a series of ordinary differential equations using a step-wise modeling approach to characterize the experimental data. In the first step, the cell cycle base model was constructed with saturation of cell numbers and contact inhibition of cell proliferation included to describe the vehicle control data. In the second step, the cell cycle base model was extended to include estimates of the cytostatic and cytotoxic effects of the single drugs. Model components for drug- and cell line-specific behaviors over time and concentration were included. Finally, the cell cycle models of gemcitabine and trabectedin were combined, and combination drug effects were incorporated in the model. Parameters estimated from previous steps were fixed when performing model fitting in the subsequent steps.

### Cell cycle base model

A cell cycle base model (Figure 1A; Hamed and Roth, 2011; Hamed et al., 2013) was used previously to characterize the time course of three cell cycle phases in the absence of drug:

(1) $$\begin{array}{lll} \frac{dG_{1}}{dt} & = & 2k_{3}G_{2}-k_{1}G_{1}\;G_{1}\left(0\right)=G_{1}^{0} \end{array}$$

(2) $$\begin{array}{lll} \frac{dS}{dt} & = & k_{1}G_{1}\;-k_{2}S\;S\left(0\right)=S^{0} \end{array}$$

(3) $$\begin{array}{lll} \frac{dG_{2}}{dt} & = & k_{2}S-k_{3}G_{2\;}G_{2}\left(0\right)=G_{2}^{0} \end{array}$$

(4) $$\begin{array}{lll} R_{tot} & = & G_{1}+S+G_{2} \end{array}$$

where *G*_1_ is the number of cells in *G*_0_/*G*_1_ phase, *S* is the cell number in *S* phase, and *G*_2_ are cells in *G*_2_/*M* phase. In our study, neither *G*_0_ and *G*_1_ nor *G*_2_ and *M* were distinguishable experimentally. The total cell number is *R*_*tot*_ and the first-order transition rates among the consecutive phases are *k*_1_, *k*_2_ and *k*_3_. Equations (1)–(3) are a linear system and all phases have exponential behavior without saturation. The doubling time can be approximated by *T*_*d*_ = 1/*k*_1_ + 1/*k*_2_ + 1/*k*_3_.

Saturation of total cell growth toward a maximal cell count at steady-state *R*_*ss*_ was introduced by slowing the doubling process in Equation (1) via:

(5) $$\begin{array}{l} \frac{dG_{1}}{dt}\;=2k_{3}G_{2}\left(1-\frac{R_{tot}}{{2R}_{ss}}\right)-k_{1}G_{1}\;G_{1}\left(0\right)\;=\;G_{1}^{0} \end{array}$$

with this modification *T*_*d*_ holds only for early times. Please note that multiplication of *R*_*ss*_ by 2 is necessary to adjust for the doubling factor at *G2*, see Appendix for details.

To account for cell contact inhibition, the outflow from *G*_0_/*G*_1_ was modified by slowing the transition rate *k*_1_. The final cell cycle model (Figure 1B) without drug effects is then:

(6) $$\begin{array}{l} \frac{dG_{1}}{dt}=\;2k_{3}G_{2}\left(\text{1}-\frac{R_{tot}}{\text{2}R_{ss}}\right)\;-\;k_{1}\left(\text{1}-\frac{I_{max}t^{\gamma }}{IT_{50}^{\gamma }+t^{\gamma }}\right)G_{1}\\ \;G_{1}(0)=G_{1}^{\;0} \end{array}$$

(7) $$\begin{array}{lll} \frac{dS}{dt} & = & k_{1}\left(1-\frac{I_{max}t^{\gamma }}{IT_{50}^{\gamma }+t^{\gamma }}\right)G_{1}-k_{2}S\;S\left(0\right)=S^{0} \end{array}$$

(8) $$\begin{array}{lll} \frac{dG_{2}}{dt} & = & k_{2}S\;-k_{3}G_{2}\;G_{2}\left(0\right)=G_{2}^{0} \end{array}$$

(9) $$\begin{array}{lll} R_{tot} & = & G_{1}+S+G_{2} \end{array}$$

where *I*_*max*_ is the maximal growth inhibition effect of cell contact, *IT*_50_ is the time when the half-maximal inhibition effect is achieved, and γ is the Hill coefficient. For increasing times, the states of the three cell phases in Equations (6)–(9) converge toward:

(10) $$\begin{array}{lll} G_{1}^{*} & = & \frac{R_{ss}}{1+\frac{k_{1}}{k_{2}}\left(1-I_{max}\right)+\frac{k_{1}}{k_{3}}\left(1-I_{max}\right)}, \end{array}$$

(11) $$\begin{array}{lll} S^{*} & = & \frac{k_{1}}{k_{2}}{\left(1-I_{max}\right)G}_{1}^{*}\;and\;G_{2}^{*}=\frac{k_{1}}{k_{3}}{\left(1-I_{max}\right)G}_{1}^{*}. \end{array}$$

The Appendix provides derivations of Equations (10) and (11).

### Cell cycle model of gemcitabine and trabectedin as single agents

Gemcitabine and trabectedin have concentration- and time-dependent effects on the transition rates. In addition, the MiaPaCa-2 and BxPC-3 cell lines showed different behaviors, and appropriate model adjustments were necessary for these factors. To avoid repetitive representation of the specific model equations, we present a general model structure and list only the necessary adjustments for the different drugs and cell lines. The general model is:

(12) $$\begin{array}{l} \frac{dG_{1}}{dt}\;=\;2k_{3}e_{Z,3}(C_{Gem},C_{Tbd})G_{2}(1-\frac{R_{tot}}{2R_{ss}})\;G_{1}(0)=G_{1}^{\;0\;}\\ \;-\;k_{1}e_{Z,\text{1}}(C_{Gem},C_{Tbd})(\text{1}-\frac{I_{max}t^{\gamma }}{IT_{50}^{\gamma }+t^{\gamma }})G_{1}\\ \;-\;a_{Gem}(i_{G1}^{Gem})G_{1}-a_{Tbd}(i_{G1}^{Tbd})G_{1} \end{array}$$

(13) $$\begin{array}{l} \frac{dS}{dt}=k_{1}e_{Z,\text{1}}(C_{Gem},C_{Tbd})(\text{1}-\frac{I_{max}t^{\gamma }}{IT_{50}^{\gamma }+t^{\gamma }})G_{1}\;S(0)=S^{\;0\;}\\ \;-\;k_{2}e_{Z,\text{2}}(C_{Gem},C_{Tbd})S\\ \;-\;a_{Gem}(i_{S}^{Gem})S-a_{Tbd}(i_{S}^{Tbd})S \end{array}$$

(14) $$\begin{array}{l} \frac{dG_{2}}{dt}=k_{2}e_{Z,\text{2}}(C_{Gem},C_{Tbd})S-k_{3}e_{Z,\text{3}}(C_{Gem},C_{Tbd})G_{2}\\ \;G_{2}(0)=G_{2}^{\;0\;}\\ \;-\;a_{Gem}(i_{G2}^{Gem})G_{2}-a_{Tbd}(i_{G2}^{Tbd})G_{2} \end{array}$$

(15) $$\begin{array}{l} \frac{dA}{dt}=a_{Gem}(i_{G\text{1}}^{Gem})G_{1}+a_{Tbd}(i_{G\text{1}}^{Tbd})G_{1}+a_{Gem}(i_{S}^{Gem})S\\ \;+a_{Tbd}(i_{S}^{Tbd})S\;+\;a_{Gem}(i_{G\text{2}}^{Gem})G_{2}+a_{Tbd}(i_{G\text{2}}^{Tbd})G_{2}\\ \;-k_{apop}A\;A(\text{0})\text{ = 0} \end{array}$$

(16) $$R_{tot}=G_{1}+S+G_{2}+A$$

where *C*_*Gem*_ and *C*_*Tbd*_ are the concentrations of gemcitabine and trabectedin, *A* is the cell number in the *sub G*_1_ phase, and *k*_*apop*_ is the first-order rate constant for apoptosis. Drug and cell-line effects on the transition rates are modeled by the effect functions *e*_*Z*, 1_, *e*_*Z*, 2_, and *e*_*Z*, 3_ for gemcitabine and trabectedin, where *Z* denotes either the MiaPaCa-2 (M) or BxPC-3 (B) cell line. Apoptotic drug effects are described by on/off functions of the form:

(17) $$a_{X}(i)=\{\begin{array}{c} K_{\;4,X}\;if\;i=1\\ 0\;if\;i=0 \end{array}$$

where *i* = 1 indicates that a delayed apoptotic effect occurs as described by:

(18) $$K_{X}\;=\frac{K_{max,\;X}C_{X}^{\gamma_{X}}}{KC_{\;50,\;X}^{\gamma_{X}}+C_{X}^{\gamma_{X}}}$$

(19) $$\frac{dK_{1,\;X}}{dt}=\frac{1}{\tau_{X}}(K_{X}-K_{\text{1},\;X})\;K_{\text{1},\;X}(0)\text{=0}$$

(20) $$\frac{dK_{j,\;X}}{dt}=\frac{1}{\tau_{X}}(K_{j\;-\;1,X}-K_{j,\;X})K_{j,\;X}(0)\;=0\;j\;=\;2,\ldots ,4$$

where *K*_*X*_ represents the non-linear cytotoxicity function, *K*_*j, X*_ are transit steps, *K*_*max, X*_ is the maximum killing rate, *KC*_50, *X*_ is the sensitivity constant, and γ_*X*_ is the Hill coefficient. *X* indicates either *Gem* or *Tbd*. If no apoptotic effect exists, *i* = 0 in Equation (17). Four transit steps were previously found (Miao et al., 2016a) optimal to describe the time delay.

### Gemcitabine

The general cell cycle models structure described above was refined to characterize gemcitabine effects on MiaPaCa-2 (Figure 1C) and BxPC-3 (Figure 1D) cells. Gemcitabine-induced cell cycle arrest was modeled as inhibition of the transition rate between *S* and *G*2/*M* phase. It was assumed that gemcitabine induced apoptosis from *S* phase for MiaPaCa-2 cells and both *G*_0_/*G*_1_, and *S* phases for BxPC-3 cells. This accounts for the greater sensitivity of BxPC-3 cells to gemcitabine compared to MiaPaCa-2 cells (Miao et al., 2016a).

#### Model equations for gemcitabine on MiaPaCa-2 cells

The transition rate *k*_1_ increases with gemcitabine concentrations as described by:

(21) $$\begin{array}{l} e_{M,1}\left(C_{Gem},0\right)=\exp\left(\alpha_{k1}\left(C_{Gem}-C_{ref}\right)\right) \end{array}$$

where α_*k*1_ is a rate constant, and *C*_*ref*_ is a reference concentration. In this study, *C*_*ref*_ is always set to the lowest gemcitabine concentration in all subsequent terms. As a result, *k*_1_ will increase exponentially with increasing gemcitabine concentrations. Gemcitabine-induced cell cycle arrest in *S* phase is modeled by inhibition of transition rate *k*_2_ with:

(22) $$\begin{array}{l} e_{M,2}\left(C_{Gem},0\right)\\ =\left(1-\frac{I_{max,Gem}{\left(1-\exp\left(-k\left(C_{Gem}-C_{ref}\right)\right)\right)C}_{Gem}^{\gamma_{Gem1}}}{{IC}_{50,Gem}^{\gamma_{Gem1}}+C_{Gem}^{\gamma_{Gem1}}}\right)\; \end{array}$$

where *I*_*max, Gem*_ represents gemcitabine maximum inhibition in *S* phase, *IC*_50, *Gem*_ is the gemcitabine concentration inducing 50% of cell cycle arrest, and _γ_*Gem*_1_ is the Hill coefficient. Of note, the cell cycle arrest in *S* phase is concentration-dependent, and thus an exponential term with rate of *k* is used to describe increased inhibition as gemcitabine concentrations increase. *C*_*ref*_ is always set to the lowest gemcitabine concentration. The *C*_*ref*_ was used for gemcitabine not for trabectedin. This is because the concentration-response curve for gemcitabine is more gradual compared to trabectedin, and the gemcitabine concentrations were chosen at about 0, $\frac{1}{2}IC_{50}$, *IC*_50_ and 2*IC*_50_ values for both cell lines, thus the concentration-dependency is more prominent at high concentrations for gemcitabine. For trabectedin, the concentration-response curve is steeper, so the concentration-dependency is more prominent and there is no need to incorporate *C*_*ref*_. The exponential term has values between 0 and 1. The transition rate *k*_3_ is not affected by time and concentration, and we set:

(23) $$\begin{array}{l} e_{M,3}\left(C_{Gem},0\right)\;=\;1. \end{array}$$

Apoptotic effects are assumed to occur in the *S* phase only, and therefore we have $i_{G1}^{Gem}\;=\;i_{G2}^{Gem}\;=\;0$, $i_{S}^{Gem}\;=\;1$ and $i_{G1}^{Tbd}\;=\;i_{S}^{Tbd}\;=\;i_{G2}^{Tbd}\;=\;0$. The overall model utilizes Equations (12)–(20) with Equations (21)–(23), and the schematic is depicted in Figure 1C.

#### Model equations for gemcitabine on BxPC-3 cells

No effect of gemcitabine was observed on *k*_1_ and we set

(24) $$\begin{array}{l} e_{B,1}\left(C_{Gem},0\right)\;=\;1. \end{array}$$

For early time points all drug-exposed cells are arrested in *S* phase. But this effect wanes with longer exposure times. Therefore, Equation (22) is extended by an exponential term, and the time- and concentration-dependent effect of gemcitabine on *k*_2_ is given by:

(25) $$\begin{array}{l} e_{B,2}\left(C_{Gem},0\right)\;=\;\left(1-\exp\left(-\alpha_{k2}t\right)\right)\\ \left(1-\frac{I_{max,Gem}\left(1-\exp\left(-k\left(C_{Gem}-C_{ref}\right)\right)\right)C_{Gem}^{\gamma_{Gem1}}}{{IC}_{50,Gem}^{\gamma_{Gem1}}\;+\;C_{Gem}^{\gamma_{Gem1}}}\right).\; \end{array}$$

The exponential term was applied to *k*_2_ to describe *k*_2_ increases with rate constant α_*k*2_. This is based on the assumption that gemcitabine perturbs *k*_2_ by changing cell cycle regulation such as cyclin-CDK protein expression over time (Yip-Schneider et al., 2001).

In addition, we assume that *k*_3_ increases as gemcitabine concentrations increase, as described by:

(26) $$\begin{array}{l} e_{B,3}\left(C_{Gem},0\right)\;=\text{ exp}\left(\alpha_{k3}\left(C_{Gem}-C_{ref}\right)\right). \end{array}$$

Apoptosis occurs in the *G*_1_ and *S* phases, and we set $i_{G1}^{Gem}\;=\;i_{S}^{Gem}\;=\;1$, $i_{G2}^{Gem}\;=\;0$ and $i_{G1}^{Tbd}\;=\;i_{S}^{Tbd}\;=\;i_{G2}^{Tbd}\;=\;0$. The overall model includes Equations (12)–(20) with Equations (24)–(26). The schematic is depicted in Figure 1D.

### Trabectedin

Trabectedin-induced cell cycle arrest was modeled as inhibition of the transition from *S* to *G*_2_/*M* phases and from *G*_2_/*M* to *G*_0_/*G*_1_ phases, and cells in all cycle phases may commit to apoptosis. A diagram of the cell cycle model for trabectedin is shown in Figure 1E.

#### Model equations for trabectedin on MiaPaCa-2 cells

The transition rate *k*_1_ increases with increasing trabectedin concentration by:

(27) $$\begin{array}{l} e_{M,1}\left({0,C}_{Tbd}\right)=\exp\left(\beta_{k1}C_{Tbd}\right). \end{array}$$

The transition rate *k*_2_ increases over time by:

(28) $$\begin{array}{l} e_{M,2}\left({0,C}_{Tbd}\right)\;=\;\left(1-\frac{I_{max,Tbd,S}\;\exp\left(-k_{S}t\right)C_{Tbd}^{\gamma_{Tbd_{1}}}}{IC_{50,Tbd,S}^{\gamma_{Tbd1}}+C_{Tbd}^{\gamma_{Tbd_{1}}}}\right) \end{array}$$

whereas *k*_3_ is inhibited over time:

(29) $$\begin{array}{l} e_{M,3}\left(0,C_{Tbd}\right)\;=\;\left(1-\frac{I_{max,Tbd,G2}\left(1-\exp\left(-k_{G2}t\right)\right)C_{Tbd}^{\gamma_{Tbd_{2}}}}{IC_{50,Tbd,G2}^{\gamma_{Tbd2}}\;+\;C_{Tbd}^{\gamma_{Tbd_{2}}}}\right). \end{array}$$

Additionally, apoptosis can initiate in all phases by setting $i_{G1}^{Tbd}\;=\;i_{S}^{Tbd}\;=\;i_{G2}^{Tbd}\;=\;1$ and $i_{G1}^{Gem}\;=\;i_{S}^{Gem}\;=\;i_{G2}^{Gem}\;=\;0$. The overall model entails Equations (12)–(20) with Equations (27)–(29).

#### Model equations for trabectedin on BxPC-3 cells

Transition rates *k*_1_ and *k*_2_ were modeled with Equations (27) and (28). We assumed that the inhibition of transition rate *k*_3_ increases over time and *k*_3_ exhibits concentration-dependency:

(30) $$\begin{array}{l} e_{B,3}\left({0,C}_{Tbd}\right)\;=\;\left(1-\frac{I_{max,Tbd,G2}\left(1-\exp\left(-k_{G2}t\right)\right)C_{Tbd}^{\gamma_{Tbd_{2}}}}{IC_{50,Tbd,G2}^{\gamma_{Tbd2}}\;+\;C_{Tbd}^{\gamma_{Tbd_{2}}}}\right)\\ \exp\left(\beta_{k3}C_{Tbd}\right). \end{array}$$

Apoptosis can initiate in all phases, described by $i_{G1}^{Tbd}\;=\;i_{S}^{Tbd}=i_{G2}^{Tbd}\;=\;1$ and $i_{G1}^{Gem}\;=\;i_{S}^{Gem}=i_{G2}^{Gem}\;=\;0$. The overall model includes Equations (12)–(20) with Equations (27), (28), and (30).

### Cell cycle model of drug combinations

The time- and concentration-dependent drug effects of gemcitabine and trabectedin as single agents on *k*_1_ and *k*_3_ are multiplied for the drug combination. Both drugs interact at the transition from *S* to *G*2/*M* phase and at the induction of apoptosis. Instead of multiplying the single effects, we assume competitive drug interactions for cell cycle arrest in *S* phase and apply the competitive combination effect term from Ariëns et al. (Ariens et al., 1957; Koch et al., 2016). For the induction of apoptosis, the effects of both cytotoxic drug effects are summed (Miao et al., 2016a). To account for remaining interaction effects not predicted by the model, two interaction parameters, ψ_1_ and ψ_2_ (Chakraborty and Jusko, 2002; Koch et al., 2009) were included at the points where the drugs interact on cell cycle inhibition and induction of apoptosis. If ψ_1_ or ψ_2_ equals 1, the effect of the combination is additive, based on the applied combination effect term. A ψ_1_ or ψ_2_ value smaller than 1 indicates synergistic interaction, and any value greater than 1 indicates antagonistic combination behavior.

#### Model equations for combinations on MiaPaCa-2 cells

The drug interaction on *k*_1_ was modeled with multiplication of single drug effects. For *k*_1_ we obtain:

(31) $$\begin{array}{l} e_{M,1}\left(C_{Gem},C_{Tbd}\right)=\exp\left(\alpha_{k1}\left(C_{Gem}-C_{ref}\right)\right)\exp\left(\beta_{k1}C_{Tbd}\right). \end{array}$$

Because both gemcitabine and trabectedin act on the transition from *S* to *G*_2_/*M*, we assumed a competitive behavior and multiplied the interaction parameter ψ_1_ by the *IC*_50_ of gemcitabine:

(32) $$\begin{array}{l} E\left(C_{Gem},C_{Tbd}\right)=\left(1-\frac{I_{max,Gem}\left(1-\exp\left(-k\left(C_{Gem}-C_{ref}\right)\right)\right){\left(\frac{C_{Gem}}{{\psi_{1}IC}_{50,Gem}}\right)}^{{\gamma_{Gem}}_{1}}+I_{max,Tbd,S}\;\exp\left(-k_{S}t\right){\left(\frac{C_{Tbd}}{{IC}_{50,Tbd,S}}\right)}^{\gamma_{Tbd1}}}{1+{\left(\frac{C_{Gem}}{{\psi_{1}IC}_{50,Gem}}\right)}^{{\gamma_{Gem}}_{1}}+{\left(\frac{C_{Tbd}}{{IC}_{50,Tbd,S}}\right)}^{\gamma_{Tbd1}}}\right) \end{array}$$

and set

(33) $$\begin{array}{l} e_{M,2}\left(C_{Gem},C_{Tbd}\right)\;=\;E\left(C_{Gem},C_{Tbd}\right). \end{array}$$

In Equation (18), ψ_2_ is multiplied by the *KC*_50, *Gem*_ of gemcitabine. Because gemcitabine has no effect on *k*_3_, (compare Equation 23), the final equations for the combination model are Equations (12)–(20) with Equations (29), (31)–(33) where we set $i_{G1}^{Gem}=i_{G2}^{Gem}=0$, $i_{S}^{Gem}=1$ and $i_{G1}^{Tbd}=i_{S}^{Tbd}=i_{G2}^{Tbd}=1$.

#### Model equations for combinations on BxPC-3 cells

For *k*_1_ we apply Equation (27). The transit rate *k*_2_ is influenced by the competitive effect Equations (32) and a time-dependent effect, (compare Equation 25):

(34) $$\begin{array}{l} e_{B,2}\left(C_{Gem},C_{Tbd}\right)=\left(1-\exp\left(-\alpha_{k2}t\;\right)\right)E\left(C_{Gem},C_{Tbd}\right). \end{array}$$

For *k*_3_:

(35) $$\begin{array}{l} {\;e}_{B,3}\left(C_{Gem},C_{Tbd}\right)\\ =\left(1-\frac{I_{max,Tbd,G2}{\left(1-\exp\left(-k_{G2}t\right)\right)C}_{Tbd}^{\gamma_{Tbd2}}}{{IC}_{50,Tbd,G2}^{\gamma_{Tbd2}}+C_{Tbd}^{\gamma_{Tbd2}}}\right)\\ \exp\left(\alpha_{k3}\left(C_{Gem}-C_{ref}\right)\right)\exp\left(\beta_{k3}C_{Tbd}\right). \end{array}$$

The model equations are Equations (12)–(20) with Equations (27), (32), (34), (35) and $i_{G1}^{Gem}\;=\;i_{S}^{Gem}\;=\;1$, $i_{G2}^{Gem}\;=\;0$, $i_{G1}^{Tbd}\;=\;i_{S}^{Tbd}\;=\;i_{G2}^{Tbd}\;=\;1$.

### Data analysis

The modeling was performed using ADAPT 5 software (D'Argenio et al., 2009) with a naive pooled approach. Models were fitted to the data using the maximum likelihood (ML) estimation method. The variance model was:

(36) $$\begin{array}{l} V_{i}={\left(\sigma_{1}+\sigma_{2}Y_{i}\right)}^{2} \end{array}$$

where *V*_*i*_ is the variance of the *i*th time point, σ_1_ and σ_2_ are variance model parameters, and *Y*_*i*_ is the predicted response at *i*th time point. The goodness of fit was assessed by visual inspection of model fittings, goodness-of-fit plots, the Akaike Information Criteria (AIC), and the coefficients of variation (CV %).

## Results

### Cell cycle without perturbation (baseline model)

**Figure 2:**
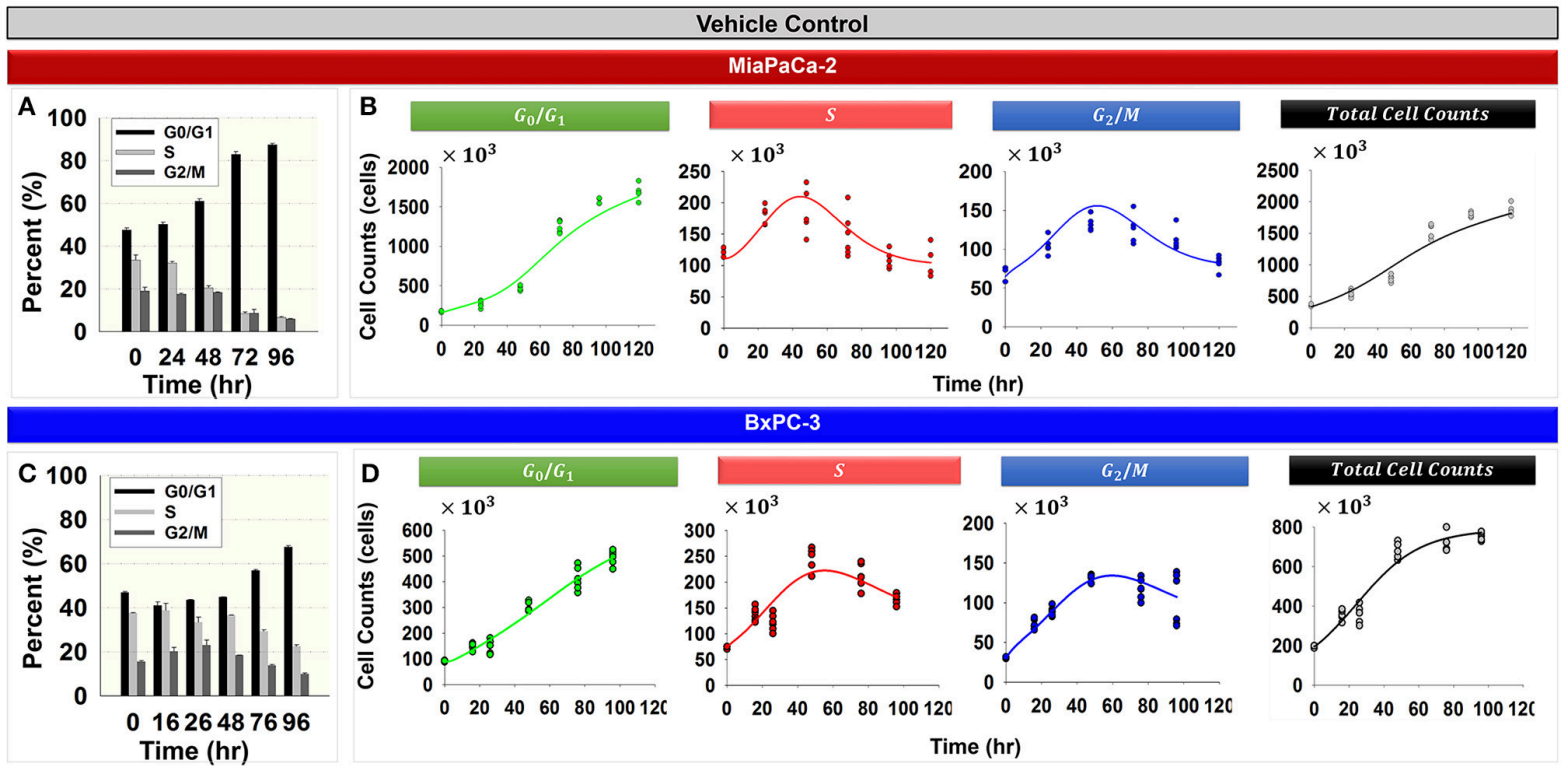
**Cell cycle phase distributions in the vehicle control groups for MiaPaCa-2 and BxPC-3 cells. (A,C)** Percent of cells in *G*_0_/*G*_1_, *S*, and *G*_2_/*M* phases at 0, 24, 48, 72, and 96 h (MiaPaCa-2) and 0, 16, 26, 48, 72, and 96 h (BxPC-3) for vehicle control groups. *Columns*, mean of triplicate; *bars*, SD. *N* = 3. **(B,D)** Data and model fitting of vehicle control groups. Numbers of cells in *G*_0_/*G*_1_, *S*, and *G*_2_/*M* phases, as well as total cells over time, are represented by symbols. Model fittings based on the model of Figure 1B are indicated by lines.

Figure 2 shows the cell cycle distribution of MiaPaCa-2 (Figures 2A,B) and BxPC-3 cells (Figures 2C,D) in the absence of drug. Both cell lines showed progressive accumulation in the *G*_0_/*G*_1_ phase with time (Figures 2A,C). Numerous factors can cause accumulation of the cell population in the *G*_0_/*G*_1_ phase. Serum starvation, a reduction in nutrients, or contact inhibition as a result of increasing cell confluence activate cell growth checkpoints and cell cycle arrest occurs at the *G*_0_/*G*_1_ phase (Hayes et al., 2005; Choresca et al., 2009; Dalman et al., 2010).

Total cell numbers and the number of cells in different cycle phases, were modeled simultaneously with the cell cycle base model (Figure 1B). The model was able to characterize well the observed data (Figures 2B,D). Parameter estimates are shown in Table 2. These model estimates were then fixed for subsequent analyses. The approximate doubling time *T*_*d*_ for MiaPaCa-2 was 31.9 and 16.8 h for BxPC-3 cells. Cells finally reached a steady-state *R*_*ss*_, which was 6.00 × 10^6^ *cells* (fixed to a value observed in the study, data not shown) for MiaPaCa-2 and 7.98 × 10^5^ *cells* for BxPC-3 cells. Under the experimental conditions used here, the time to reach half of the maximal cell contact inhibition was 45.4 h for MiaPaCa-2 and 61.0 h for BxPC-3 cells.

**Table 2:** **Pharmacodynamic model parameter estimates for two cell lines. Parameters were obtained after fitting to the cell cycle base model shown in Figure 1B**.

**Cell Line Parameters**	**Definition**	**Units**	**MiaPaCa-2 Estimate (CV%)**	**BxPC-3 Estimate (CV%)**
*k*_1_	Transition rate between *G*_0_/*G*_1_ and *S* phase	*h*^−1^	0.0693 (7.92)	0.166 (6.58)
*k*_2_	Transition rate between *S* and *G*_2_/*M* phase	*h*^−1^	0.101 (4.11)	0.149 (5.43)
*k*_3_	Transition rate between *G*_2_/*M* and *G*_0_/*G*_1_ phase	*h*^−1^	0.132 (3.99)	0.245 (5.78)
$G_{1}^{0}$	Initial cell number at *G*_0_/*G*_1_ phase	*cells*×10^3^	159 (5.30)	91.6 (4.60)
*S*^0^	Initial cell number at *S* phase	*cells*×10^3^	111 (5.55)	73.5 (4.75)
$G_{2}^{0}$	Initial cell number at *G*_2_/*M* phase	*cells*×10^3^	64.9 (5.83)	30.2 (5.04)
*R*_*ss*_	Cell number at steady-state	*cells*×10^3^	6000^a^	798 (2.75)
*I*_*max*_	Maximum inhibition on *k*_1_		0.922 (1.80)	0.868 (22.8)
*IT*_50_	Time inducing 50% of maximum inhibition *I*_*max*_	*h*	45.4 (6.31)	61.0 (14.4)
γ	Hill coefficient		4.37 (16.9)	3.30 (43.8)

a *The value is fixed*.

### Gemcitabine effects upon cell cycle and model prediction

**Figure 3:**
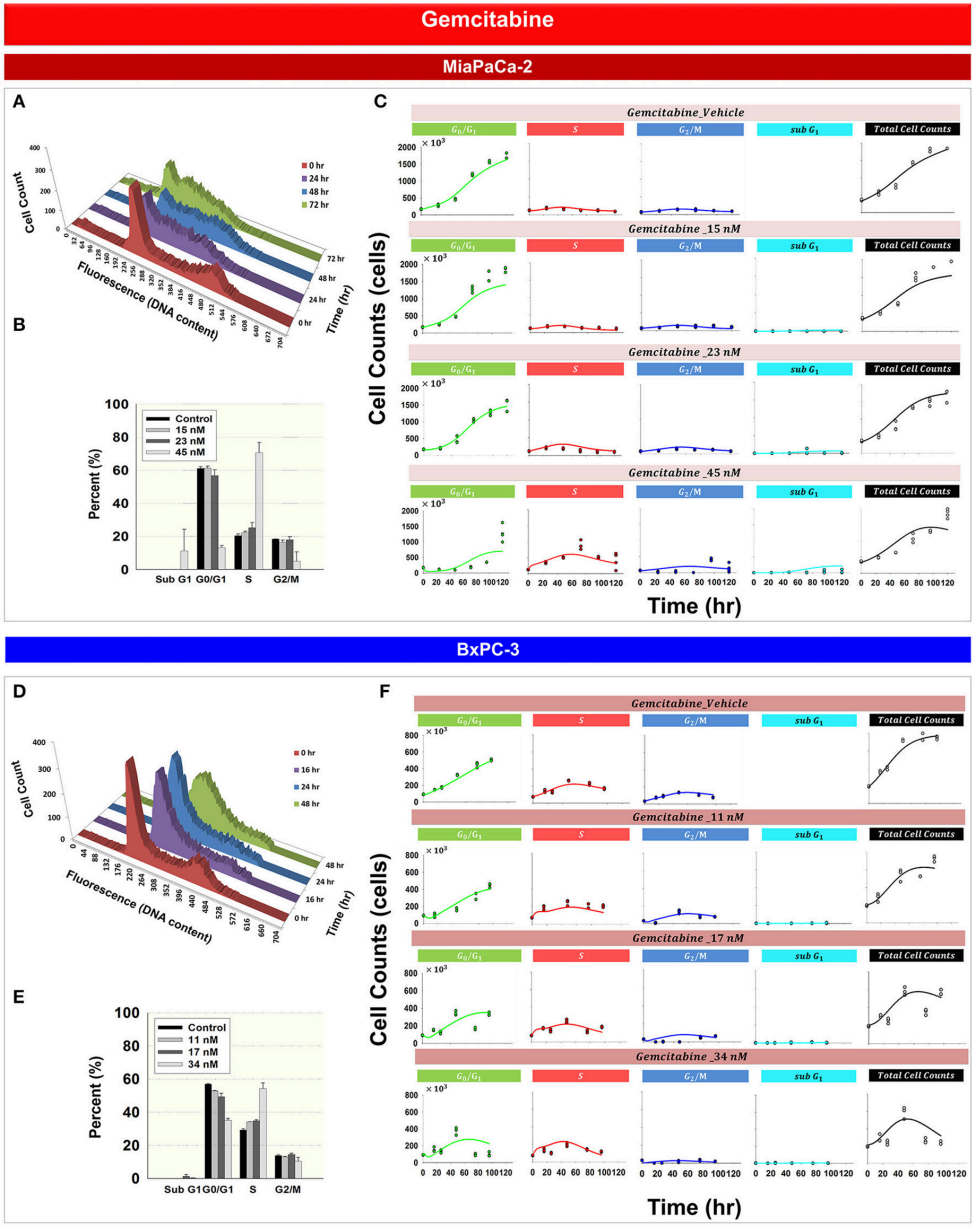
**Gemcitabine effects on the cell cycle for MiaPaCa-2 and BxPC-3 cells**. **(A,D)** Histograms showing temporal changes in cellular DNA content after incubation with 45 nM gemcitabine in MiaPaCa-2 cells and 34 nM in BxPC-3 cells. All data are in triplicate. **(B,E)** fraction of cells in *subG*_1_, *G*_0_/*G*_1_, *S*, and *G*_2_/*M* phases after incubating cells with concentrations of gemcitabine shown for 48 (MiaPaCa-2) or 76 (BxPC-3) h. *Columns*, mean of triplicate determinations; *bars*, SD. **(C,F)** Data and model fitting results of cells incubating cells with concentrations of gemcitabine shown for up to 120 h. Symbols represent data for cells numbers in *G*_0_/*G*_1_, *S*, *G*_2_/*M*, *subG*_1_ phase as well as total cell numbers; lines show model fittings based on PD models in Figure 1C (MiaPaCa-2) and Figure 1D (BxPC-3).

Figure 3 shows the effects of gemcitabine on the cell cycle distribution of MiaPaCa-2 (Figures 3A–C) and BxPC-3 cells (Figures 3D–F). As time increased, 45 nM gemcitabine induced *S* phase accumulation of MiaPaCa-2 cells (Figure 3A and Supplemental Table 1). A gradual increase of cells in *S* phase also was observed after exposure of BxPC-3 cells to 34 *nM* gemcitabine (Figure 3D and Supplemental Table 1). Gemcitabine-induced *S* phase cell cycle arrest was concentration-dependent for both MiaPaCa-2 (Figure 3B) and BxPC-3 cells (Figure 3E). Apoptosis, measured as the percentage of sub-diploid cells, appeared at the highest-tested gemcitabine concentrations for both cell lines.

Based upon mechanisms of gemcitabine effects on the cell cycle, mathematical models were developed for MiaPaCa-2 (Figure 1C) and BxPC-3 cells (Figure 1D). Figures 3C,F show the model fittings. In general, the model well captured the trend of the observed data. Considering the large number and complexities of the datasets, model performance is acceptable. Table 3 shows parameter estimates for the drugs as single agents. Gemcitabine-induced *S* phase arrest was modeled as inhibition of the transition rate from *S* to *G2/M* phase. The maximum inhibition *I*_*max, Gem*_ was fixed to 1 for both cell lines. The concentrations that induced half-maximal *S* phase arrest *IC*_50, *Gem*_ were 11.5nM for MiaPaCa-2 and 48.1 nM for BxPC-3. Because the maximal inhibition *I*_*max, Gem*_ was concentration-dependent, inhibition increased proportional to the rate constant *k*, which was 0.0281 nM^−1^ for MiaPaCa-2 and 1 nM^−1^ for BxPC-3 cells. The model assumed that gemcitabine-induced apoptosis occurred in *S* phase in MiaPaCa-2 cells, and in both *G*_0_/*G*_1_ and *S* phases in BxPC-3 cells, and the parameters related to gemcitabine killing effects were fixed to parameters derived in our previous study (Miao et al., 2016a). Minor differences between MiaPaCa-2 and BxPC-3 cells also included certain model assumptions; for MiaPaCa-2, it was assumed that *k*_1_ increased with rate of α_*k*_1__ (0.0612 *nM*^−1^) with increased gemcitabine concentrations. For BxPC-3, it was assumed that *k*_2_ and *k*_3_ increased with rates of α_*k*2_ (0.0635 *h*^−1^) and α_*k*3_(0.0605 *nM*^−1^) as gemcitabine concentrations increased.

**Table 3:** **Pharmacodynamic model parameter estimates. Parameters were obtained after fitting to cell cycle models for either gemcitabine (Figures 1C,D) or trabectedin (Figure 1E) as single agents**.

**Cell line Parameters**	**Definition**	**Units**	**MiaPaCa-2 Estimate (CV%)**	**BxPC-3 Estimate (CV%)**
*k*_*apop*_	Elimination rate from apoptosis compartment	h^−1^	0.0815 (8.71)	3.33 (17.4)
*I*_*max, Gem*_	Maximum inhibition of *k*_2_		1^a^	1^a^
*IC*_50, *Gem*_	Gemcitabine concentration inducing 50% of *I*_*max, Gem*_	nM	11.5 (264)	48.1 (12.6)
γ_*Gem*1_	Hill coefficient for gemcitabine effects on cell cycle arrest at S phase		5^a^	5^a^
*k*	Rate constant increasing *I*_*max, Gem*_ with gemcitabine concentration	nM^−1^	0.0281 (7.47)	1^a^
α_*k*1_	Rate constant ***k***_***1***_ increases with gemcitabine concentration	nM^−1^	0.0612 (6.10)	
α_*k*2_	Rate constant ***k***_***2***_ increases with time	h^−1^		0.0635 (13.7)
α_*k*3_	Rate constant ***k***_***3***_ increases with gemcitabine concentration	nM^−1^		0.0605 (8.21)
*I*_*max, Tbd, S*_	Trabectedin maximum inhibition at *S* phase arrest		1^a^	1^a^
*IC*_50, *Tbd, S*_	Trabectedin concentration inducing 50% of *I*_*max, Tbd, S*_	nM	0.222 (>100)	1.03 (4.57)
γ_*Tbd*1_	Hill coefficient for trabectedin effects at *S* phase arrest		5^a^	5^a^
*I*_*max, Tbd, G*2_	Trabectedin maxium inhibition at *G*_2_/*M* phase arrest		1^a^	1^a^
*IC*_50, *Tbd, G*2_	Trabectedin concentration inducing 50% of *I*_*max, Tbd, G*2_	nM	0.525 (5.87)	0.681 (5.64)
γ_*Tbd*2_	Hill coefficient for trabectedin effects at *G*_2_/*M* phase arrest		5^a^	5^a^
*k*_*S*_	Rate constant *I*_*max, Tbd, S*_ decreases with time	h^−1^	1^a^	0.00204 (135)
*k*_*G*2_	Rate constant *I*_*max, Tbd, G*2_ increases with time	h^−1^	0.0270 (4.32)	0.0516 (21.1)
β_*k*1_	Rate constant ***k***_***1***_ increases with trabectedin concentration	nM^−1^	2.64 (2.60)	0.637 (12.1)
β_*k*3_	Rate constant ***k***_***3***_ increases with trabectedin concentration	nM^−1^		0.296 (66.3)
*K*_*max, Gem*_	Maximal cell kill constant for gemcitabine	h^−1^	0.166^b^	0.0613^b^
*KC*_50, *Gem*_	Gemcitabine concentration inducing 50% of *K*_*max, Gem*_	nM	41.5^b^	21.4^b^
1/τ_*Gem*_	Transit constant for gemcitabine	h^−1^	0.0370 (4.00)	0.0671^b^
γ_*Gem*_	Hill coefficient for gemcitabine		0.527 (44.5)	2.08^b^
*K*_*max, Tbd*_	Maximal cell kill constant for trabectedin	h^−1^	0.0858^b^	0.261^b^
*KC*_50, *Tbd*_	Trabectedin concentration inducing 50% of *K*_*max*−*Et*743_	nM	1.63^b^	6.31^b^
1/τ_*Tbd*_	Transit constant for trabectedin	h^−1^	0.0569 (4.31)	0.0452^b^
*gamma*_*Tbd*_	Hill coefficient for trabectedin		2.43 (9.26)	1.07^b^

a *The value is fixed*.

b *The value is fixed to parameter estimates from Table 3 in Miao et al. (2016a)*.

### Trabectedin effects upon cell cycle and model prediction

**Figure 4:**
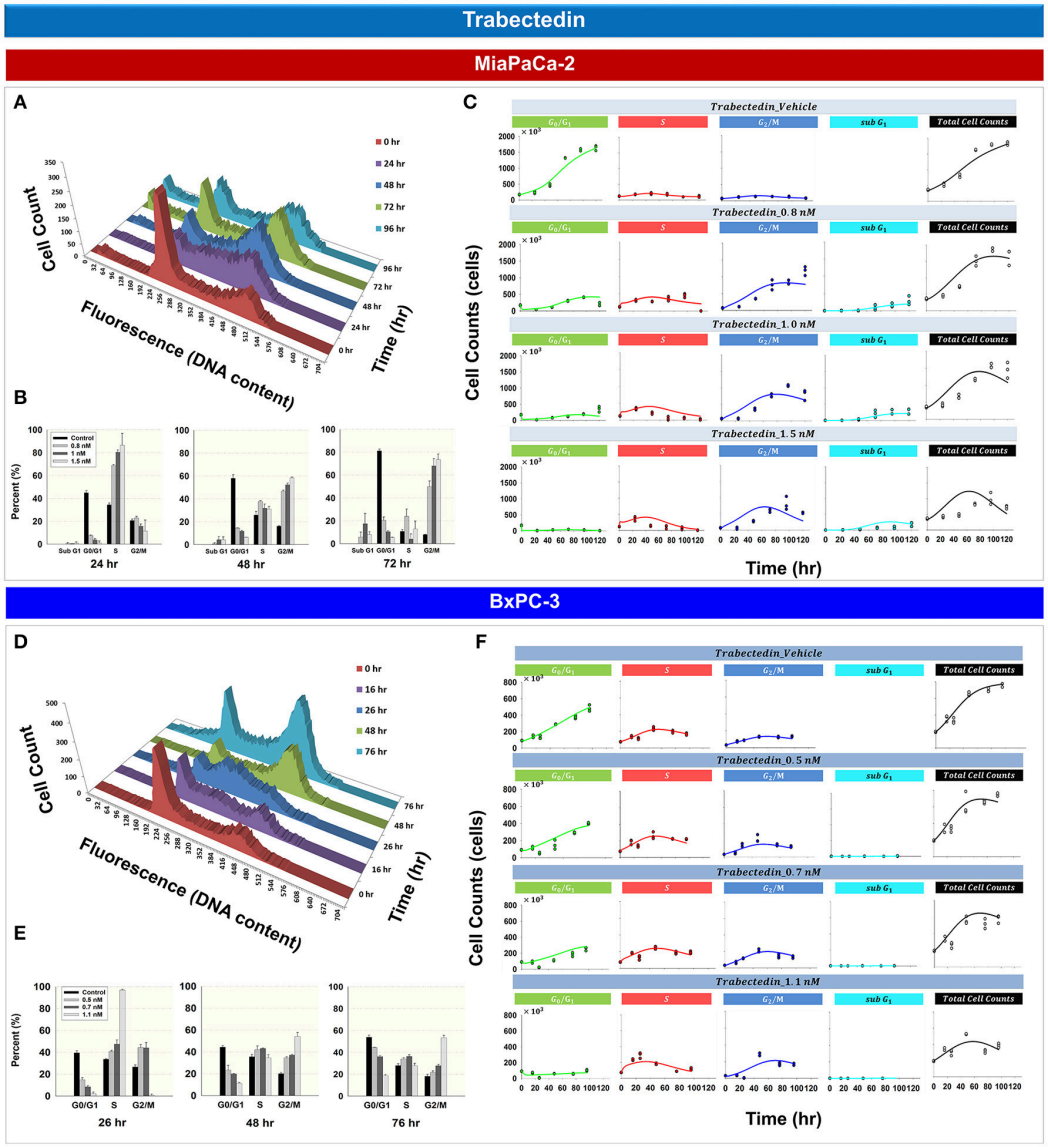
**Trabectedin effects on the cell cycle for MiaPaCa-2 and BxPC-3 cells. (A,D)** histograms showing temporal changes in cellular DNA content after continuous exposure of cells to 0.8 nM (MiaPaCa-2) or 1.1 nM (BxPC-3) trabectedin. All data are in triplicate. (**B,E)** Fraction of cells in *sub G*_1_, *G*_0_/*G*_1_, *S*, and *G*_2_/*M* phase after incubating cells in the concentrations of trabectedin shown for 24, 48, and 72 h (MiaPaCa-2) or 26, 48, and 76 h (BxPC-3). *Columns*, mean of triplicate determinations; *bars*, SD. **(C,F)** Temporal change in number of cells in *G*_0_/*G*_1_, *S*, *G*_2_/*M*, sub *G*_1_ phase, as well as total cell numbers, after incubating cells with concentrations of trabectedin shown for up to 120 (MiaPaCa-2) and 96 h (BxPC-3). Symbols represent observed data and lines represent fitted curves generated from PD model shown in Figure 1E.

Figure 4 shows the effects of trabectedin on the cell cycle distribution for MiaPaCa- 2 (Figures 4A–C) and BxPC-3 cells (Figures 4D–F). For both cell lines, trabectedin induced cell cycle arrest at *S* phase at early time points, followed by progression to *G*_2_/*M* phase at later time points. Figure 4A shows the cell cycle distribution of MiaPaCa-2 cells after exposure to 0.8 nM trabectedin for 0, 24, 48, 72, and 96 h. At 24 h, cell cycle arrest occurred in the *S* phase (Supplemental Table 1). Similar effects were observed in BxPC-3 cells (Figure 4D and Supplemental Table 1). Trabectedin effects on the cell cycle were concentration-dependent (Figures 4B,E), for both cell lines at early time points, and *S* phase accumulation increased with concentration. As exposure times increased (48–76 h) the accumulation shifted from *S* to G_2_/*M* phase.

Based upon the mechanistic effects of trabectedin on the cell cycle, mathematical models were developed (Figure 1E). Trabectedin effects on cell cycle checkpoints were modeled with inhibition effects on both *S to G*_2_/*M* phase transition *k*_2_ and the *G*_2_/*M* to *G*_0_/*G*_1_ phase transition *k*_3_. In order to characterize early *S* phase arrest followed by subsequent *G*_2_/*M* phase arrest, models were constructed in such a way that *S* phase inhibition decreased with time with rate *k*_*S*_ and inhibition in *G*_2_/*M* phase increased with time with rate *k*_*G*2_. The *k*_*S*_ was 1 h^−1^ for MiaPaCa-2 and 0.00204 h^−1^ for BxPC-3, whereas *k*_*G*2_ was 0.0270 h^−1^ for MiaPaCa-2 and 0.0516 h^−1^ for BxPC-3 cells. Trabectedin concentrations inducing half-maximal *S* phase inhibition (*IC*_50, *Tbd, S*_) were 0.222 nM for MiaPaCa-2 and 1.03 nM for BxPC-3 cells. Concentrations inducing half-maximal *G*_2_/*M* phase inhibition (*IC*_50, *Tbd, G*2_) were 0.525 nM for MiaPaCa-2 and 0.681 nM for BxPC-3 cells, suggesting that MiaPaCa-2 cells are also more sensitive to trabectedin-induced *G*_2_/*M* phase arrest than BxPC-3 cells. In addition, we also assumed concentration-dependent trabectedin effects on *k*_1_, with rates of β_*k*1_ of 2.64 nM^−1^ for MiaPaCa-2 and 0.637 nM^−1^ for BxPC-3. For BxPC-3, *k*_3_ also increased with trabectedin concentration, with a rate of β_*k*3_ of 0.296 *nM*^−1^. Finally, trabectedin-induced apoptosis was assumed to occur in all cell cycle phases. For both cell lines, the parameters related to trabectedin killing effects were fixed to values obtained previously (Miao et al., 2016a).

### Modeling combined drug effects upon cell cycle

**Figure 5:**
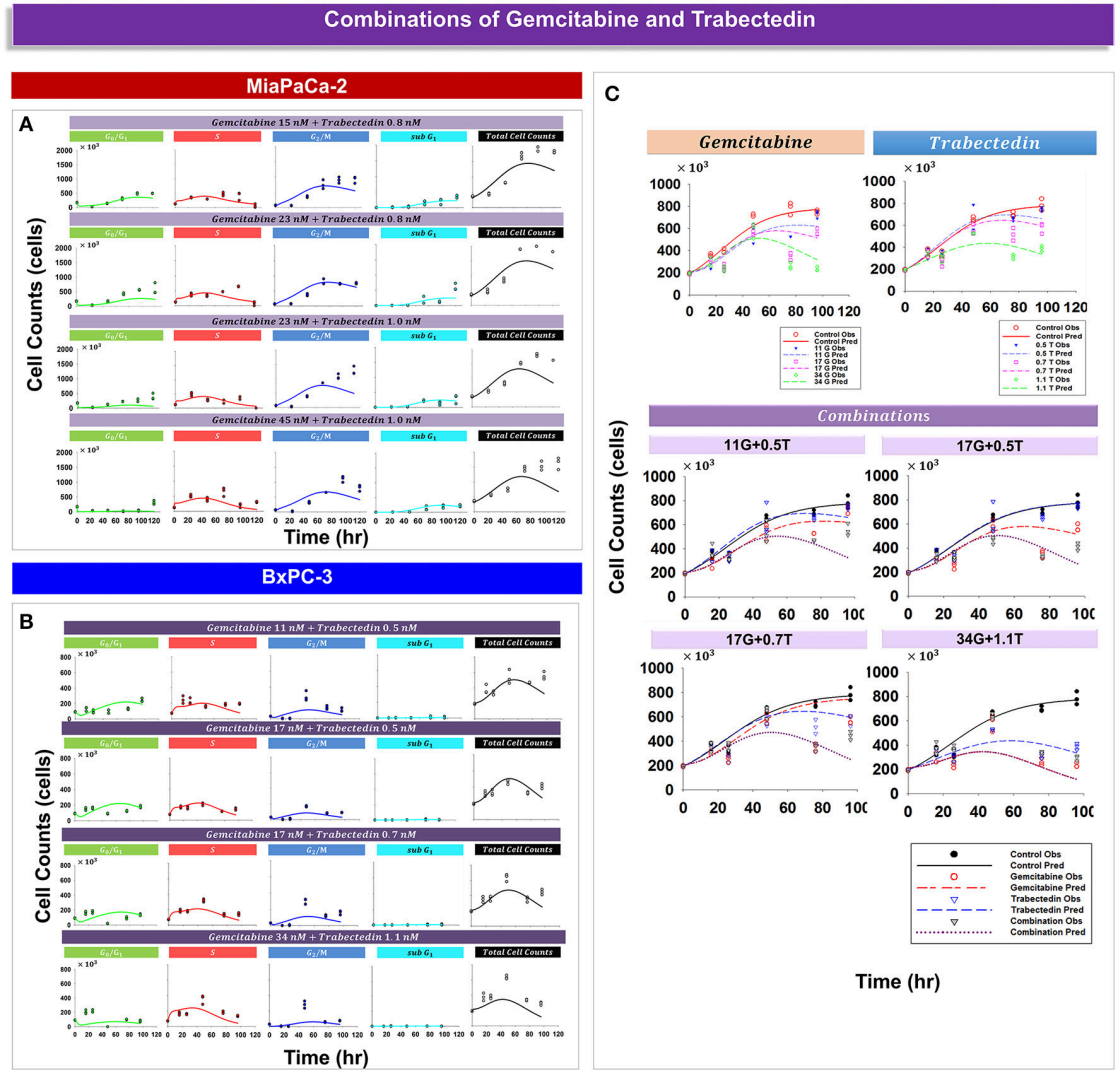
**Drug combination effects and model-fittings based on individual drug mechanisms for MiaPaCa-2 and BxPC-3 cells. (A,B)** Observed data and model fitting results for cells exposed to different combinations of drug concentrations. Symbols represent cell numbers in *G*_0_/*G*_1_, *S*, *G*_2_/*M*, and sub *G*_1_ phases; lines depict model fittings to the PD model shown in Figures 1F,G. **(C)** Shown are temporal cell proliferation profiles and model fitting results for single drug effects and four different drug combinations for total BxPC-3 cells. Symbols represent experimental data and lines indicate the corresponding model fittings.

Analysis of experimental data suggested that trabectedin and gemcitabine exert different effects upon cell cycle progression. For both cell lines, gemcitabine induced cell cycle arrest in *S* phase (Figure 3), but with trabectedin a population of cells passed through *S* phase and accumulated in *G*_2_/*M* phase cells (Figure 4). In order to model combined drug effects upon the cell cycle, the PD models for the single agents were integrated (Figures 1F,G) and used to fit simultaneously the data for four different drug concentration combinations on MiaPaCa-2 (Figure 5A) and BxPC-3 cells (Figure 5B). Figure 5C shows the BxPC-3 cell growth model fitting of experimental data for BxPC-3 cells. The model captured the trend of the data well.

Table 4 provides parameter estimates and statistics for the combined drugs. For both cell lines, a competitive interaction equation was used to model *S* phase inhibition by the two drugs. The interaction term ψ_1_ was multiplied at *IC*_50, *Gem*_. The ψ_1_ interaction parameter was fixed to 1 for MiaPaCa-2 cells (because modeling of ψ_1_ for *S* phase inhibition resulted in a value of about 1) and estimated as 0.975 for BxPC-3 cells, suggesting additive drug interaction on *S* phase inhibition. The ψ_2_ was multiplied by *KC*_50, *Gem*_ to characterize drug interactions for induction of apoptosis. Estimates of ψ_2_ were 0.499 for MiaPaCa-2 cells and 0.363 for BxPC-3 cells, indicating synergistic interactions in inducing apoptosis.

**Table 4:** **Pharmacodynamic interaction model parameter estimates**.

**Parameters**	**Definition**	**MiaPaCa-2 Estimates (CV%) 95% CI**	**BxPC-3 Estimates (CV%) 95%CI**
**ψ_1_**	Interaction term for *S* phase inhibition	1^a^	0.975 (35.9) 0.275–1.675
**ψ_2_**	Interaction term for induction of apoptosis	0.499 (74.5) 0−1.243	0.363 (16.8) 0.241–0.485

a *The value is fixed*.

*Parameters were obtained after fitting to cell cycle models for combined drug interaction effects (Figures 1F,G)*.

## Discussion

Mathematical models serve to help integrate information from complex studies, assess mechanisms of drug interactions, and guide selection and optimization of drug combinations. We previously introduced a mathematical framework based on a semi mechanism-based approach to model gemcitabine and trabectedin combination effects on pancreatic cancer cells (Miao et al., 2016a). That simple and reasonable approach could be applied to other drugs, and could also be useful to characterize time- and concentration-dependent drug-drug interactions. The present work goes beyond such semi-mechanistic models. It incorporates cell subpopulation information and enlarges the previous model in order to characterize drug combination effects of gemcitabine and trabectedin on cell cycle distribution.

Cancer cells are heterogeneous and asynchronous populations are composed of cell subpopulations in different cycle phases (Evan and Vousden, 2001). Drug sensitivity or mechanism may vary for cells in different cycle phases. Cell cycle arrest at different phases could influence cell sensitivity or resistance to drugs significantly and affect overall responses to single or multiple chemotherapeutic agents. To account for such drug effects, we developed four cell cycle models: a cell cycle base model lacking drug effects, cell cycle models for gemcitabine and trabectedin as single agents, and a cell cycle model for drug combinations. The models were applied to large experimental datasets that captured relevant pharmacodynamic endpoints such as cell proliferation, cell cycle phase, and induction of apoptosis. A step-wise modeling approach was used, first fitting the control (vehicle) data to the cell cycle base model (Figure 2), followed by fitting single drug effects to the cell cycle models of gemcitabine (Figure 3) and trabectedin (Figure 4). Ultimately, the combined data were fitted with a cell cycle model of the drug combination (Figure 5).

The total cell number was measured during drug exposure, as was the fraction of cells in each cycle phase (*G*_0_/*G*_1_, *S*, *G*_2_/*M*) and in apoptosis (*sub G*_1_). The developed model incorporates not only changes in cell cycle distribution, but also the dynamics of the whole cell population. The dataset was large, consisting of ~500 samples that each provided data for 5 cell populations, and was analyzed using ordinary differential equations.

The proposed cell cycle model framework was developed based on drug mechanisms observed in the study. For the vehicle-treated controls, the data show that the *G*_0_/*G*_1_ fraction increased with time, reflecting the fact that when cells reach confluence, contact inhibition occurs, nutrients may become limited, and a cell growth checkpoint is activated toward the end of *G*_1_ phase. If cells are able to progress to *S* phase, normal cycling occurs. Otherwise, cells enter a resting *G*_0_ phase until they are able to resume cycling. For the vehicle control group, the logistic growth function was used to characterize saturation of total cells, while contact inhibition was used to characterize *G*_0_/*G*_1_ phase cells increasing over time. The contact inhibition was modeled with a time-dependent Hill function. We have also tested contact inhibition with a feedback loop where the inhibition of *k*_1_ is dependent on *R*_*tot*_. However, the time-dependent Hill type of equation was chosen as it allows us to estimate the time where the half-maximal effects of cell contact inhibition occurs and to simplify the convergence analysis as presented in the Appendix. Our model for gemcitabine was initially premised on that of Hamed et al. (2013), but more extensive measurements provided clearer insights into drug effects on apoptosis. Further, for gemcitabine, time- and concentration-dependent cell cycle arrest occurred at the *S* phase, consistent with previous results (Yip-Schneider et al., 2001; Morgan et al., 2005). The gemcitabine-induced *S* phase arrest was modeled as both an increase in the transition rate from *G*_0_/*G*_1_ and an inhibition of transition rates from *S* phase to *G*_2_/*M* phase. The *S* phase arrest increased with gemcitabine concentrations. Therefore, gemcitabine-induced cell death is modeled with a non-linear killing function, with the delay of killing effects modeled by four transit compartments. Trabectedin induced cell cycle arrest in *S* phase at earlier time points, but at later times, cells progressed through *S* to *G*_2_/*M* phase. In order to capture the time-dependency, equations were constructed such that an inhibition term on the transition from *S* to *G*_2_/*M* decreased with time, and the inhibition term on the transition from *G*_2_/*M* to *G*_0_/*G*_1_ increased with time. In addition, we assumed that cells in all three cell cycle phases can commit to apoptosis after drug treatment. The models employed a similar basic structure for both MiaPaCa-2 and BxPC-3 cells, with only minor changes required to reflect the unique characteristics of the specific cell line. For example gemcitabine induced apoptosis in *S* phase for MiaPaCa-2 cells, but apoptosis occurred in BxPC-3 cells in both *S* and *G*_0_/*G*_1_ phases. The models were constructed with differences between cell lines. For example, based on the *KC*_50_ of the two cell lines (Miao et al., 2016a), MiaPaCa-2 is more trabectedin-sensitive while BxPC-3 is more gemcitabine-sensitive. In addition, the two cells lines also have phenotypic and genotypic differences; for example, MiaPaCa-2 has mutant K-ras and p53 genes, whereas BxPC-3 has wild-type K-ras and p53 genes (Deer et al., 2010). The final model permitted a higher-resolution investigation of the basis of the synergistic interaction of these drugs than previously (Miao et al., 2016a). An interaction term ψ was incorporated into specific mechanistic model components to explore model behavior when interactions in those mechanisms were hypothesized to explain observed drug effects of the combination that exceeded model predictions. In the final developed model, ψ_1_ was applied to represent drug interactions at the point of *S* phase arrest, and ψ_2_ was applied to represent drug interactions at the point of induction of apoptosis. However, ψ_1_ was approximately equal to 1, suggesting that drug interactions in inducing *S* phase accumulation were additive, whereas ψ_2_ was <1, suggesting that drug interactions in inducing apoptosis were synergistic.

Cell-cycle-specific compartmental models have been applied previously for investigation of cancer chemotherapy agents. Simple models can be constructed with as few as 4-5 compartments that represent each cycle phase (Kozusko et al., 2001; Florian et al., 2005; Ribba et al., 2009). Compartments can be added to represent apoptosis or other death mechanisms (Basse et al., 2004; Panetta et al., 2006; Sherer et al., 2006; Zhu et al., 2015), and additional components may be included to account for more complex pharmacological mechanisms such as cell cycle regulation (Senderowicz, 2004; Ferrell et al., 2011). Most existing cell cycle models characterize single drug effects; few reports model drug combinations (Gardner, 2002; Zhu et al., 2015). Because of the layers of complexities in larger and richer data sets, there is a need for models to account for those complexities. The cell cycle models developed here allow investigation of the specific effects of the individual drugs in combination therapy upon cell cycle progression. For example, we identified that gemcitabine exerts influence in the *S* phase, whereas trabectedin exerts influence in both *S* and *G*_2_/*M* phases. In addition, the models have the flexibility to account for conditions under which drug effects upon specific cycle phases or phase transitions are concentration- or time-dependent. For example, the degree of gemcitabine-induced *S* phase arrest increased with concentration. Finally, the developed model was able to account for cell line differences; the two cell lines investigated are different in phenotype and genotype (Deer et al., 2010), and in their sensitivity to gemcitabine and trabectedin (Miao et al., 2016a). Model development showed that to capture differences in cell line–specific behaviors, drug effects may be hypothesized to affect different, or even multiple cycle phase transition rates, and apoptosis may be induced from different cell cycle phases.

Traditional approaches to model tumor growth often include a logistic or Gompertzian growth model. Such models are empirical and do not take into account complex biological mechanisms such as the cell cycle dynamics of subpopulations. Here we used models based upon cell cycle structure to describe cancer cell growth and drug effects. The final model characterized very well not only cell cycle dynamics, but also cancer cell proliferation (Figure 5C). The model can characterize cancer cell growth without drug perturbation, and growth in response to single- or combined cell-cycle-specific agents.

In conclusion, we have modeled chemotherapeutic drug effects of single and combined agents on pancreatic cancer cell cycle dynamics and apoptosis. The study suggests a basis for the overall gemcitabine/trabectedin synergy observed previously (Miao et al., 2016a) when examines cell cycle subpopulations. The proposed model structure is not limited to gemcitabine and trabectedin as single or combined agents, and can be adapted to investigate efficacy of other cell-cycle specific agents. Even though the cell cycle models developed in this study are used to characterize *in vitro* data, the model structures may be kept and integrated with more model components accounting for cell and tissue heterogeneity when translating to *in vivo* work. However, the mechanisms underlying synergy within specific cell subpopulations remains undefined at the level of protein- and pathway interactions. Further research, employing approaches such as high-resolution proteomic investigation of drug action, may provide the means to integrate information at that scale into the model structure proposed here.

The model components and complications that were introduced in this report for the dual drug effects on MiaPaCa-2 cells have been largely confirmed in our further studies that utilized proteomic and western blot methods to assess drug effects on various signaling pathways (Miao et al., 2016b). The present report reflects the second part of our multi-stage efforts to perform studies that employ modeling to assess relevance of diverse physiologic and pharmacologic processes and then employ more sophisticated methodology to delve into possible and identified mechanisms and complexities.

## Author contributions

Participated in research design: XM and WJJ. Conducted experiments: XM. Performed data analysis: XM and GK. Wrote or contributed to the writing of the manuscript: XM, GK, SAO, RMS, WJJ.

## Funding

This work was supported by National Institutes of Health Grants 24211 to WJJ and CA168454 and CA198096 to RMS, the National Research Fund, Luxembourg to GK, and co-funded under the Marie Curie Actions of the European Commission (FP7-COFUND).

### Conflict of interest statement

The authors declare that the research was conducted in the absence of any commercial or financial relationships that could be construed as a potential conflict of interest.

## Abbreviations

CCM: cell cycle model
DDI: drug-drug interaction
PK/PD: pharmacokinetics/pharmacodynamics.

## Appendix

### Behavior of cell cycle model with saturation for increasing time

For *t* → ∞ we obtain from Equations (6)–(8):

(A1) $$\begin{array}{lll} 0 & = & 2k_{3}G_{2}^{*}\left(1-\frac{G_{1}^{*}+S^{*}+G_{2}^{*}}{2R_{ss}}\right)-k_{1}\left(1-I_{max}\right)G_{1}^{*} \end{array}$$

(A2) $$\begin{array}{lll} 0 & = & k_{1}\left(1-I_{max}\right)G_{1}^{*}-k_{2}S^{*} \end{array}$$

(A3) $$\begin{array}{lll} 0 & = & k_{2}S^{*}-k_{3}G_{2}^{*}. \end{array}$$

From Equation (A3) one finds:

(A4) $$\begin{array}{l} S^{*}=\frac{k_{3}}{k_{2}}G_{2}^{*}. \end{array}$$

From Equations (A2) and (A4) we have:

(A5) $$\begin{array}{l} G_{2}^{*}=\frac{k_{1}\left(1-I_{max}\right)}{k_{3}}G_{1}^{*}. \end{array}$$

Hence, with Equation (A5) we obtain:

(A6) $$\begin{array}{l} S^{*}=\frac{k_{1}}{k_{2}}\left(1-I_{max}\right)G_{1}^{*}. \end{array}$$

Substituting Equations (A5) and (A6) in (A1) results with *b* = 1−*I*_*max*_ in:

$$\begin{array}{l} 0=2k_{1}bG_{1}^{*}\left(1-\frac{G_{1}^{*}\left(1+\frac{k_{1}}{k_{2}}b+\frac{k_{1}}{k_{3}}b\right)}{2R_{ss}}\right)-k_{1}bG_{1}^{*}. \end{array}$$

We set $a=1+\frac{k_{1}}{k_{2}}b+\frac{k_{1}}{k_{3}}b$, and cancel out the zero-solution to obtain:

(A7) $$\begin{array}{l} 0=1-\frac{G_{1}^{*}a}{R_{ss}}\;\Leftrightarrow \;G_{1}^{*}=\frac{R_{ss}}{a} \end{array}$$

yielding Equations (10) and (11). Summation of $G_{1}^{*}$, *S*^*^ and $G_{2}^{*}$ from Equations (10) and (11) fulfills $R_{tot}^{*}=R_{ss}^{*}$.
