# Supplementary material for: Pharmacodynamic Modeling of Cell Cycle Effects for Gemcitabine and Trabectedin Combinations in Pancreatic Cancer Cells
Source: Front Pharmacol. 2016 Nov 15;7:421. doi: 10.3389/fphar.2016.00421 (PMC5108803; doi:10.3389/fphar.2016.00421)
Supplement: Supplementary file 1 [file Presentation1.pdf]

Supplementary information

Pharmacodynamic Modeling of Cell Cycle Effects for Gemcitabine and Trabectedin Combinations in Pancreatic Cancer Cells

Authors: Xin Miao[1], Gilbert Koch[1,3], Sihem Ait-Oudhia[2], Robert M. Straubinger[1] and William J. Jusko[1*]

[1]Department of Pharmaceutical Sciences, University at Buffalo, SUNY, Buffalo, New York;

[2]Center for Pharmacometrics and Systems Pharmacology (Orlando), Department of Pharmaceutics, College of Pharmacy, University of Florida, Florida;

[3]Pediatric Pharmacology and Pharmacometrics, University of Basel, Children's Hospital, Basel, Switzerland

Running title: Drug combination cell cycle models

Corresponding author:

Dr. William J. Jusko

wjjusko@buffalo.edu

Table 1. The raw data for Figure 3A, D and Figure 4A, D. Cell cycle was analyzed by the ModFit LT software as the percentage of cells in each cell cycle phase after DNA staining with PI.

**MiaPaCa-2 (45 nM Gemcitabine)**			
**Time (h)**	G_0/G_1 **(%)**	S **(%)**	G_2/M **(%)**
0	47.33	35.55	17.12
0	48.63	30.72	20.65
0	46.81	33.89	19.31
24	24.38	66.65	8.97
24	24.19	71.88	3.93
24	22.66	77.31	0.03
48	15.44	83.33	1.23
48	14.42	76.45	9.14
72	19.12	80.74	0.14
72	18.85	81.15	0.00
72	13.90	86.10	0.00
**BxPC-3 (34 nM Gemcitabine)**			
**Time (h)**	G_0/G_1 **(%)**	S **(%)**	G_2/M **(%)**
0	46.92	37.36	15.72
0	47.34	37.58	15.08
0	46.38	37.69	15.92
16	54.68	45.28	0.04
16	54.94	45.06	0.00
16	51.35	48.65	0.00
26	59.91	40.09	0.00
26	58.33	41.67	0.00
26	54.52	45.48	0.00
48	63.85	34.60	1.55
48	63.20	32.37	4.43
48	64.53	34.28	1.19
76	36.37	50.49	13.14
76	34.35	56.36	9.29
76	35.07	56.17	8.76

Table 1. Continued

MiaPaCa-2 (0.8 nM Trabectedin)			
**Time (h)**	G_0/G_1 (%)	S (%)	G_2/M (%)
0	47.33	35.55	17.12
0	48.63	30.72	20.65
0	46.81	33.89	19.31
24	7.36	70.58	22.07
24	7.89	67.97	24.14
24	7.76	68.99	23.25
48	14.51	37.85	47.64
48	14.82	38.70	46.49
48	14.73	37.67	47.60
72	23.46	31.16	45.38
72	22.21	24.45	53.34
72	19.40	19.81	60.80
96	25.40	24.52	50.07
96	24.45	29.44	46.11
96	23.35	26.16	50.49
**BxPC-3 (1.1 nM Trabectedin)**			
**Time (h)**	G_0/G_1 (%)	S (%)	G_2/M (%)
0	46.92	37.36	15.72
0	47.34	37.58	15.08
0	46.38	37.69	15.92
16	19.87	68.19	11.94
16	21.56	69.26	9.18
16	21.09	68.30	10.61
26	3.74	96.26	0.00
26	2.12	97.40	0.48
26	0.83	97.28	1.89
48	11.72	36.68	51.6
48	10.85	32.48	56.67
76	18.36	25.79	55.85
76	19.65	27.97	52.38
76	18.15	29.90	51.95
